# A High-Throughput System for Cyclic Stretching of Precision-Cut Lung Slices During Acute Cigarette Smoke Extract Exposure

**DOI:** 10.3389/fphys.2020.00566

**Published:** 2020-06-05

**Authors:** Jarred R. Mondoñedo, Elizabeth Bartolák-Suki, Samer Bou Jawde, Kara Nelson, Kun Cao, Adam Sonnenberg, Walter Patrick Obrochta, Jasmin Imsirovic, Sumati Ram-Mohan, Ramaswamy Krishnan, Béla Suki

**Affiliations:** ^1^Department of Biomedical Engineering, College of Engineering, Boston University, Boston, MA, United States; ^2^Boston University School of Medicine, Boston, MA, United States; ^3^Department of Systems Engineering, College of Engineering, Boston University, Boston, MA, United States; ^4^Department of Emergency Medicine, Beth Israel Deaconess Medical Center, Harvard Medical School, Boston, MA, United States

**Keywords:** stretcher, IL-1b, MMP-1, mechanotrasduction, emphysema

## Abstract

**Rationale:**

Precision-cut lung slices (PCLSs) are a valuable tool in studying tissue responses to an acute exposure; however, cyclic stretching may be necessary to recapitulate physiologic, tidal breathing conditions.

**Objectives:**

To develop a multi-well stretcher and characterize the PCLS response following acute exposure to cigarette smoke extract (CSE).

**Methods:**

A 12-well stretching device was designed, built, and calibrated. PCLS were obtained from male Sprague-Dawley rats (*N* = 10) and assigned to one of three groups: 0% (unstretched), 5% peak-to-peak amplitude (low-stretch), and 5% peak-to-peak amplitude superimposed on 10% static stretch (high-stretch). Lung slices were cyclically stretched for 12 h with or without CSE in the media. Levels of Interleukin-1β (IL-1β), matrix metalloproteinase (MMP)-1 and its tissue inhibitor (TIMP1), and membrane type-MMP (MT1-MMP) were assessed via western blot from tissue homogenate.

**Results:**

The stretcher system produced nearly identical normal Lagrangian strains (*E*_*xx*_ and *E*_*yy*_, *p* > 0.999) with negligible shear strain (*E*_*xy*_ < 0.0005) and low intra-well variability 0.127 ± 0.073%. CSE dose response curve was well characterized by a four-parameter logistic model (*R*^2^ = 0.893), yielding an IC_50_ value of 0.018 cig/mL. Cyclic stretching for 12 h did not decrease PCLS viability. Two-way ANOVA detected a significant interaction between CSE and stretch pattern for IL-1β (*p* = 0.017), MMP-1, TIMP1, and MT1-MMP (*p* < 0.001).

**Conclusion:**

This platform is capable of high-throughput testing of an acute exposure under tightly-regulated, cyclic stretching conditions. We conclude that the acute mechano-inflammatory response to CSE exhibits complex, stretch-dependence in the PCLS.

## Introduction

Precision-cut lung slices (PCLSs) have emerged as a valuable tool in lung biology (Tepper et al., 2005; Henjakovic et al., 2008; Khan et al., 2010; Lavoie et al., 2012; Schlepütz et al., 2012; Rosner et al., 2014; Hiorns et al., 2016; Van Dijk et al., 2016). A key advantage of this preparation is that the PCLS can be acutely exposed to disease-modifying conditions, such as enzymatic parenchymal digestion in emphysema (Van Dijk et al., 2016), while recording corresponding structural and functional changes in both space and time (Hiorns et al., 2016; Lavoie et al., 2012). PCLSs also benefit by preserving the native extracellular environment (Sanderson, 2011) and retaining nearly all of the resident cell types in the lung. These technical advantages have thus promoted widespread adoption of the PCLS in models of exposure assessment (Langer et al., 2012; Lauenstein et al., 2014; Uhl et al., 2015; Hess et al., 2016; Watson et al., 2016; Neuhaus et al., 2017), pharmacologic therapy (Switalla et al., 2010; van Rijt et al., 2015; Donovan et al., 2016; Kistemaker et al., 2017), and disease modeling, including chronic obstructive pulmonary disease (COPD) (Chronic Obstructive Lung Disease [COLD], 2017).

The overwhelming majority of this prior work has examined the PCLS under static conditions. However, the lung is continuously and rhythmically stretched during tidal breathing *in vivo* and thus, a more accurate recapitulation of native lung responsiveness demands similar dynamic conditions be imposed *ex vivo* (Suki et al., 2013). For example, the absence of stretch has been shown to influence cellular and enzymatic maintenance of tissue properties (Yi et al., 2016; Jesudason et al., 2010) by impacting the biological phenomenon known as mechanotransduction (Ingber, 2006). One of the few models incorporating cyclic stretching of PCLS showed that stretch magnitude in ventilator induced lung injury (VILI) modulated the nuclear translocation of NF-κB and oxidative stress responses in lung slices (Song et al., 2016; Davidovich et al., 2013b). It has been suggested that analogous mechanisms could facilitate emphysema progression in the lung via stretch-dependent secretion of pro-inflammatory cytokines and enzymes accelerating matrix turnover (Suki et al., 2013). Yet, comparable and potentially transformative studies aimed at elucidating the possible role of mechanotransduction in COPD pathogenesis and progression are lacking.

Here, we report the design and implementation of a multi-well equibiaxial device to cyclically stretch PCLSs obtained from excised rat lungs. Its primary advantages include high-throughput, low variance, and the ability to deliver complex, user-defined stretch patterns to the entire slice. To demonstrate the feasibility of this system in studying the mechano-inflammatory response to an acute pharmacologic exposure, we use cigarette smoke extract (CSE) during cyclic stretching to mimic cigarette smoking *in vivo*. We hypothesize the corresponding physiological response is stretch-pattern dependent. To test this, we first confirm tissue viability in this system and then compare the effects of stretch and CSE exposure on biochemical changes in several molecular markers known to play a role in COPD.

## Materials and Methods

### Device Design

**FIGURE 1:**
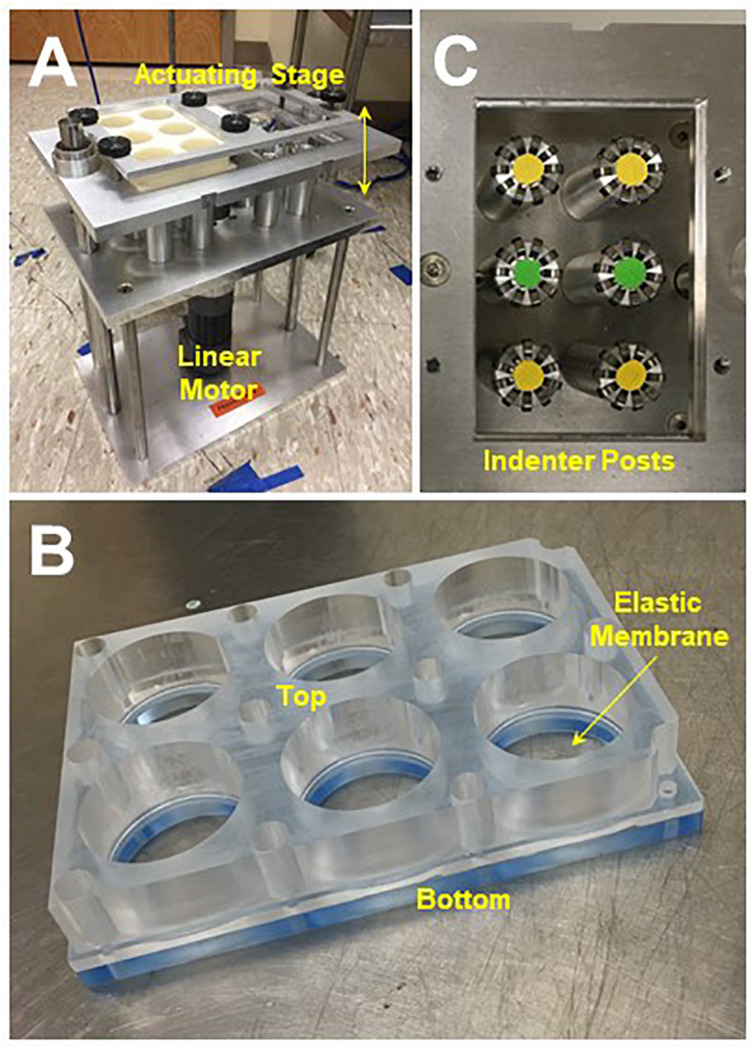
**(A)** Multi-well device for cyclic stretching of precision-cut lung slices (PCLSs), see text for design details. **(B)** Reusable 6-well flexframe with interchangeable elastic membrane. **(C)** Ball bearings affixed to the indenter posts minimized friction during stretch.

The multi-well stretching system pictured in Figure 1 was built and calibrated based on previous designs (Arold et al., 2009; Imsirovic et al., 2015). Briefly, one or two 6-well plates with deformable elastic membranes are secured in the upper stage of the stretcher. A linear actuating motor (A1 Series: Servo Cylinder, Ultra Motion, Cutchogue, NY, United States) is used to move the stage vertically. As the stage moves down, the elastic membrane in each well is stretched around a fixed, cylindrical indenter post. As the stage moves back up, the elastic membrane relaxes to its initial configuration. Cyclic stretching is achieved by repeating this process at a prescribed rate and displacement depth, which corresponds to the area strain translated to the elastic membrane. Ball bearings (McMaster-Carr, Elhmhurst, IL, United States) affixed to the top of the indenter posts reduce friction, heat generation, and hysteresis. Detailed designs available by request.

A custom software interface (Embarcadero C++ Developer, Austin, TX, United States) was developed to prescribe any simple or complex stretch pattern with parameters including waveform type, frequency, amplitude, and duration. The entire system was constructed from stainless steel and could be moved to a cell culture incubator for stretching under controlled, sterile conditions.

We also designed and fabricated a lightweight, reusable 6-well plate acrylic “flexframe” with an interchangeable elastic, silicone membrane (Specialty Manufacturing, Inc., Saginaw, MI, United States), which we validated by comparison with a commercially available alternative (BioFlex^®^ Culture Plates, Flexcell International Corp., Burlington, NC, United States). The top and bottom components of the flexframe are separable, allowing for replacement of the elastic membrane between experiments.

### Device Calibration

To calibrate the relationship between stage displacement and membrane surface area, colored acrylic markers (Pēbēo, Cedex, France) were adhered to the membrane in a circular arrangement and then tracked during quasi-static stretch to compute local radial area change. The corresponding area strain-displacement curve was used to calibrate the stretcher and prescribe area strains for cyclic stretching. Delaunay triangulation and radial displacement of individual beads were used to calculate the Lagrangian strain E_*ij*_ of the elastic membrane during stretch according to the following relation (Holzapfel, 2000):

$$d\,s^{2}-d\,s_{0}^{2}=2\,{\text{E}}_{\text{ij}}\,d\,a_{i}\,d\,a_{j}$$

where *ds* and *ds*_0_ are the segment lengths before and after deformation, respectively, of each triangle, while *da*_*i*_ and *da*_*j*_ are the changes in position of the bead vertices. To assess whether repeated stretch induced plastic deformation of the elastic membrane, this calibration procedure was repeated following 12 h of stretch.

### Animal Protocol

Protocol #16-025 was reviewed and approved by the Boston University Institutional Animal Care and Use Committee. Male Sprague-Dawley rats (*N* = 10) with body weight 343.8 ± 60.2 g were sedated via intraperitoneal injection of xylazine (10 mg/kg) and ketamine (90 mg/kg). After ensuring appropriate depth of anesthesia and analgesia, animals were euthanized via abdominal aortic exsanguination. The lungs were excised and insufflated via tracheostomy with 10–12 mL of 1.5% low melt agarose (HyAgarose, ACTGene Inc., Piscataway, NJ, United States) in Hanks’ buffered salt solution (HBSS, Sigma) at 37°C, according to previous techniques (Watson et al., 2016). Excised lungs were then placed on ice for 15 min to allow for solidification of the agarose.

### Precision-Cut Lung Slices (PCLSs)

Lung lobes were separated, trimmed to fit the tissue stage, and then sliced in cooled HBSS with thickness ∼500 μm using a vibratome (752M Vibroslice, Campden Instruments Ltd., United Kingdom). The vibratome tissue stage was modified to include an adjustable, cylindrical sleeve that was filled with agarose to help stabilize the lung lobe during slicing. PCLSs were then “punched” using either a 6 or 10 mm coring tool (Acuderm Inc., Fort Lauderdale, FL, United States) to generate round, symmetric slices. Punching the tissues after slicing the entire lobe was found to yield a greater amount of material compared to coring the lung lobes prior to slicing. PCLSs were then moved to Dulbecco’s Modified Eagle’s Medium (DMEM, Gibco) supplemented with penicillin, streptomycin, and amphotericin B (Antimycotic-Antibiotic, Gibco). To facilitate removal of residual agarose and other cellular debris, media was changed every 30 min for 0–2 h after slicing, 1 h for 2–4 h, 2 h for 4–8 h, and 24 h thereafter, similar to previous methods (Davidovich et al., 2013a, b; Song et al., 2016). Lung slices were incubated under standard conditions (5% CO_2_ at 37°C) and allowed to recover overnight.

### MTS Assay

PCLS viability was assessed via MTS assay, which is a colorimetric measure of cell metabolic activity (Berridge et al., 2005). The formazan product yielded by this reaction is proportional to the number of metabolically healthy or active cells and is quantified by measuring the absorbance at 490 nm. The colorimetric MTS assay was used according to manufacturer’s specifications. Lung slices (6 mm) were incubated in individual wells with 20 μL of MTS reagent in 200 μL of HBSS for 1.5 h at 37°C. The supernatant was then removed to a 96-well plate for measurement of optical density.

### Preparation and Potency of Cigarette Smoke Extract (CSE)

Cigarette smoke extract solutions were prepared fresh by bubbling two cigarettes (Marlboro Red, Philip Morris USA, Richmond, VA, United States) with the filters removed, through 20 mL of DMEM at a rate of 1.0 L/min to yield a stock solution of 0.1 cig/mL. Next, the solutions were sterile filtered using a 0.22 μm pore size membrane vacuum filtration system (Steriflip, EMD Millipore) to remove large tobacco debris and other small particles. To determine the CSE dose response curve, the stock solution was diluted and 6 mm lung slices (*N* = 93) were incubated in 6-well plates for 12 h with CSE concentrations ranging from 0.001 to 0.050 cig/mL (∼3 slices per 3 mL of solution in each well). Following incubation, individual slices were rinsed with warmed HBSS to remove any residual solution containing the CSE-media mix. PCLS were transferred to a 96-well plate for assessment of viability via MTS assay as described above.

### Experimental Protocol

**FIGURE 2:**
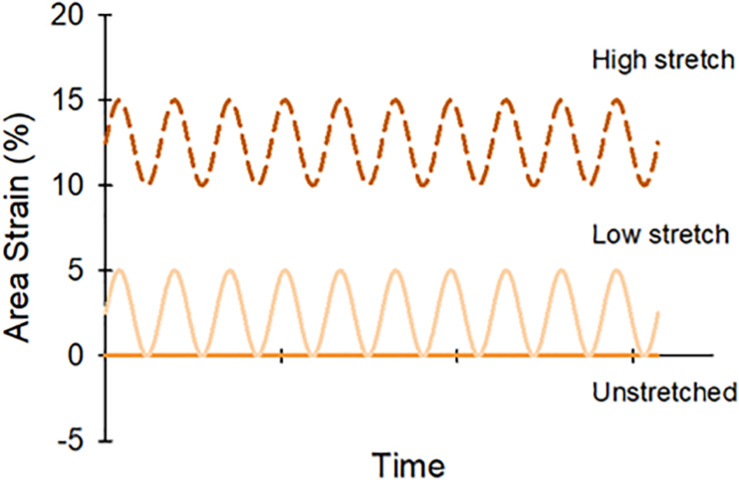
Lung slices were randomly assigned to one of three stretch patterns: unstretched (0%), low stretch (0–5% area strain), or high stretch (10–15% area strain).

Individual lung slices were attached to the center of the elastic membranes in each well using four evenly spaced beads of cyanoacrylate glue along the tissue perimeter. Initial pilot studies confirmed appropriate local tissue stretch with this preparation (Supplementary Figure S1). PCLSs were covered with 3 mL of media with or without CSE (0.01 cig/mL) for the treated and control groups, respectively, then sinusoidally stretched for 12 h at 1 Hz under standard incubation conditions. To assess the effect of different stretch patterns, PCLS were randomly assigned to one of the following three stretch amplitude groups: unstretched (US); low-stretch (LS), 5% peak-to-peak amplitude with no static stretch; and high-stretch (HS), 5% peak-to-peak amplitude superimposed on 10% static stretch. These waveforms were arbitrarily selected to simulate regions of lung experiencing different stretch during tidal breathing; a schematic is shown in Figure 2. Following cyclic stretch, the PCLSs were collected from each well for biochemical analysis (*N* = 48). Protease inhibitors EDTA and Halt Protease Inhibitor cocktail (Thermo Scientific) were added to the homogenized tissue samples, then stored at −20°C until further use. PCLSs were also collected to assess tissue viability after stretching (*N* = 49). Lung slices were trimmed using a 6 mm coring tool to reduce edge effects from slicing and the attachment procedure, then evaluated via MTS as before.

### Western Blot

Protein concentrations for the homogenized tissues were determined using the BCA colorimetric protein assay kit (Pierce, Thermo Scientific). The assay was used according to manufacturer’s specifications. Equal amounts of protein (∼3.7 μg) from each sample were separated via sodium dodecyl sulfate polyacrylamide gel electrophoresis (SDS-PAGE), transferred to a polyvinylidene difluoride (PVDF) membrane, and blocked using 5% bovine serum albumin in phosphate buffered saline containing 0.05% Tween 20 (PBS-T). All groups were run on the same membrane. After blocking for 2 h, the membrane was incubated for 1 h at room temperature with primary antibodies anti-IL-1β (1:250, Abcam), anti-MMP-1 (1:1000, Thermo Fisher Scientific), TIMP1 (1:1000, Abcam), MT1-MMP (1:5000, Abcam), and anti-GAPDH (loading control, 1 μg/ml, Abcam), washed in PBS-T 4 × 15 min, incubated with secondary antibody (anti-mouse, 1:7000, anti-rabbit, 1:10000, Vector Laboratories) for 1 h, and again washed in PBS-T 4 × 15 min. Quantitative densitometry was performed after chemiluminescence detection (SuperSignal West Pico Chemiluminescent Substrate, Pierce) with picomolar sensitivity similar to that of ELISA, with corrections for background and loading control.

### Statistical Analysis

Data analysis and fitting were performed using MATLAB (R2016a, MathWorks, Natick, MA, United States) and SigmaPlot (SigmaPlot v12.3, Systat Software, Inc., San Jose, CA, United States). CSE dose response data was fitted using a four-parameter logistic regression as follows:

$$y=a+\frac{b-a}{1+{\left(\frac{x}{c}\right)}^{d}}$$

where *y* is normalized absorbance; *x* is CSE concentration *a* and *b* are the minimum and maximum values possible, respectively, *c* is the point of inflection; and *d* is a coefficient characterizing the slope of the curve. Two-Way analysis of variance (ANOVA) was used to evaluate the influence of stretch and CSE on PCLS viability as well as on IL-1β, MT1-MMP, MMP-1, and TIMP1. Holm-Sidak method was used for *post hoc* comparisons. For all, *p* < 0.05 was considered significant.

## Results

**FIGURE 3:**
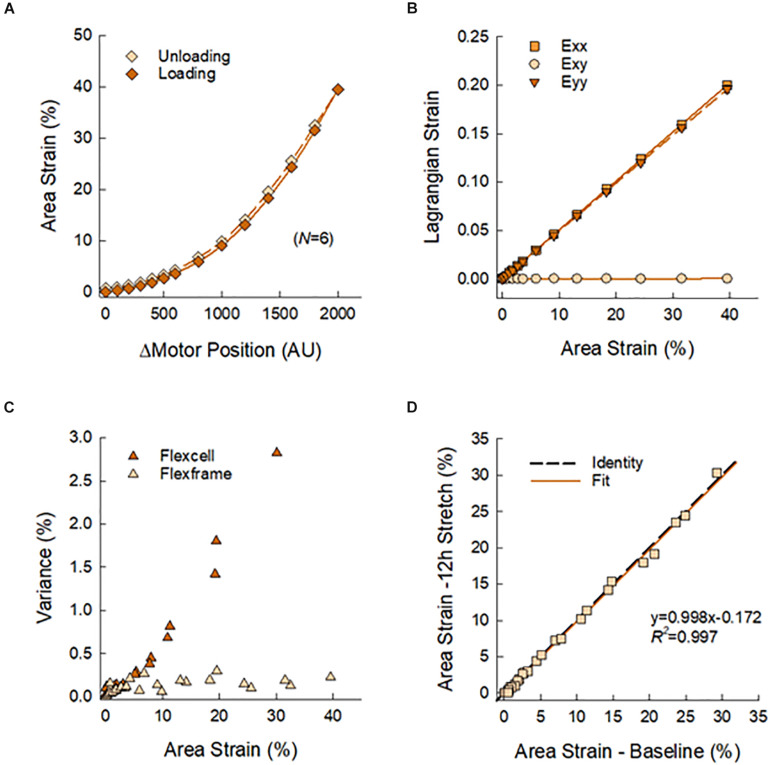
**(A)** Calibration curve used to prescribe membrane area strain as a function of motor position. Symbols represent means of *N* = 6 wells with standard deviations smaller than symbol sizes. **(B)** Nearly identical normal strains, E_xx_ and E_yy_, and negligible shear strain, E_xy_, as estimated by Delaunay triangulation, confirmed equbiaxial strain of the elastic membrane. **(C)** The reusable flexframe exhibited lower variance of area strain in comparison to a commercially available disposable alternative. **(D)** There was no observable mechanical change in the elastic membrane after 12 h of cyclic stretching.

Figure 3 presents the calibration and validation of the multi-well stretcher and FlexFrame devices. Vertical displacement of the actuating stage yielded a non-linear relation between area strain and motor position (Figure 3A), which was used to prescribe waveforms for cyclic stretching. Note the minimal hysteresis between loading and unloading of the flexframe elastic membrane, 6.34%. Normal Lagrangian strains, *E*_*xx*_ and *E*_*yy*_, were nearly identical, ρ > 0.999, with negligible shear strain, *E*_*xy*_ < 0.0005, demonstrating equibiaxial strain of the elastic membrane (Figure 3B). Compared to the commercially available BioFlex Culture Plates, our custom fabricated flexframe demonstrated lower intra-well variance for area strain, 0.473 ± 0.717% vs. 0.127 ± 0.073% (Variance Mean ± SD), particularly at larger prescribed strains (Figure 3C). Finally, there was no detectable plastic deformation of the membrane due to stretch as there was no difference in measured area strains before and after 12 h of cyclic stretching (slope: 0.998 with *R*^2^ = 0.997; Figure 3D).

**FIGURE 4:**
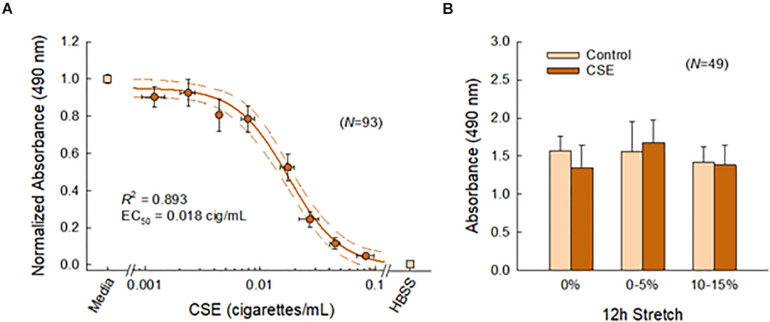
**(A)** Lung slice (*N* = 93) viability decreased with CSE concentration and was well characterized (*R*^2^ = 0.893) by a four-parameter logistic curve (solid line) shown with 95% confidence intervals (dashed lines). Binned data across multiple CSE concentrations are shown, vertical and horizontal error bars represent SE and SD, respectively. **(B)** The sub-toxic CSE concentration was selected to be 0.01 cig/mL. Two-way ANOVA detected no effects for stretch and CSE at this concentration, indicating tissue viability was not compromised with this system. Error bars represent SD.

Figure 4 shows the effects of CSE and cyclic stretch on tissue viability. We first established a sub-toxic concentration mimicking acute cigarette smoke exposure *in vivo* (Figure 4A). As expected, PCLS viability decreased with CSE concentration. The corresponding dose response curve was well characterized by a four-parameter logistic model (*R*^2^ = 0.893), yielding an IC_50_ value of 0.018 cig/mL corresponding to the CSE concentration at half-maximal viability. Based on this curve, the CSE concentration was selected to be 0.01 cig/mL for all subsequent experiments. We then confirmed tissue viability following 12 h of cyclic stretching ± CSE (Figure 4B). Two-way ANOVA detected no statistical difference in PCLS viability among different stretch patterns (*p* = 0.070), CSE exposure (*p* = 0.594), or their interaction (*p* = 0.277).

**FIGURE 5:**
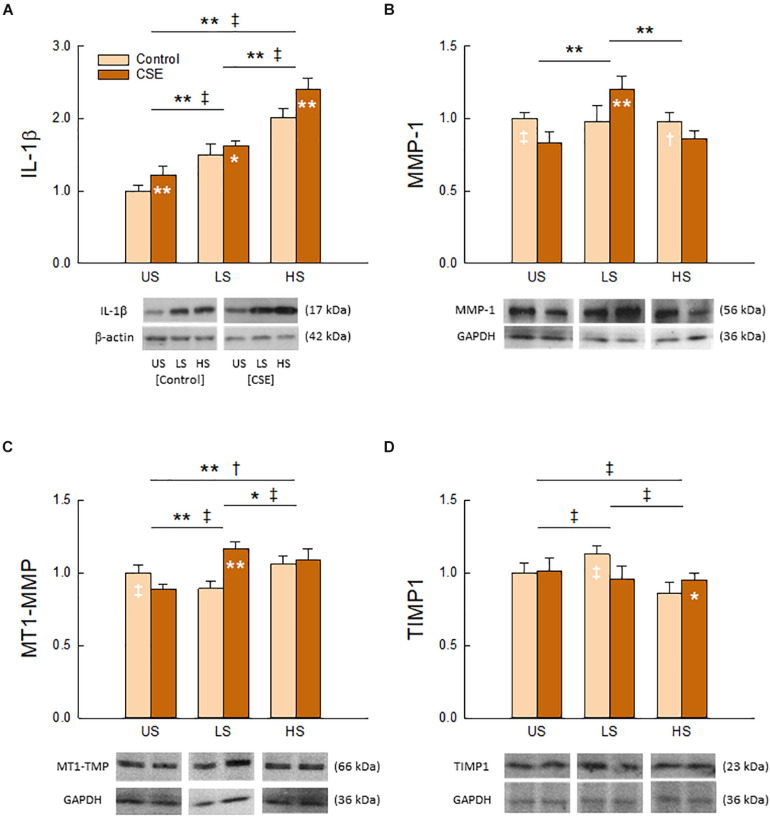
Effects of stretch pattern, CSE, and their interaction on tissue content of IL-1β **(A)**, MMP-1 **(B)**, MT1-MMP **(C)**, and TIMP1 **(D)**. Representative bands with loading controls are also shown. Data (*N* = 8) are shown as normalized mean and SD (*^†^*p* < 0.05 and **^‡^*p* < 0.001 for CSE and Control groups, respectively). For presentation purposes the original images were cut to smaller ones including representative bands in the desired order and, according to standard publication guideless, white spaces were left between them.

As shown in Figure 5, Two-Way ANOVA detected a significant interaction between stretch pattern and CSE exposure on the tissue content of all measured molecular markers (IL-1β, *p* = 0.017; MT1-MMP, MMP-1, TIMP1, *p* < 0.001). Each had a unique response to stretch and CSE. We found that IL-1β (Figure 5A) exhibited the greatest response to stretching (*p* < 0.001) among the group, and was statistically higher with CSE exposure (*p* < 0.001) for all stretch patterns. CSE also had a significant effect (*p* < 0.001) on MT1-MMP (Figure 5C), though regulation directionality depended on stretch pattern (*p* < 0.001). In contrast, stretch pattern had a significant effect on MMP-1 (Figure 5B) in the presence of CSE (*p* < 0.001), whereas it only had an effect on TIMP1 (Figure 5D) in the absence of CSE (*p* < 0.001). The enzymes MT1-MMP and MMP-1 had the greatest tissue content for LS with CSE exposure, while the tissue content of the inhibitor TIMP1 was the greatest for the same stretch pattern when CSE was absent.

## Discussion

In this study, we present the design and implementation of a multi-well stretcher to investigate the mechano-inflammatory response in lung tissue following an acute pharmacologic insult. This is the first report to combine CSE exposure with cyclic PCLS stretching as an *ex vivo* model of the dynamic changes in lung volume that occur during cigarette smoke inhalation *in vivo*. First, we demonstrated this device delivered repeatable, low-variance, equibiaxial stretch. We then characterized the CSE dose response curve in PCLSs and confirmed that cyclic stretching did not compromise tissue viability. Finally, we found the interaction between stretch pattern and CSE exposure had a significant effect on all of the molecular markers, with each exhibiting a unique response pattern. Together, these findings demonstrate the feasibility of using this system to recapitulate tidal breathing-like conditions in PCLS, while identifying specific stretch-dependent molecular responses to acute CSE exposure.

Various approaches have been reported for stretching PCLS. Techniques range from suturing (Davidovich et al., 2013a, b) or clamping (Dassow et al., 2010) the PCLS to a deformable elastic membrane, to compressing it between a polyacrylamide gel and a hollow indenter (Lavoie et al., 2012). While such devices allow for real-time imaging, they can be time consuming, limited to a single lung slice, or constrained to a small area-of-stretch. In contrast, the multi-well device described here provides simultaneous, whole-slice stretching of up to 12 samples with minimal preparation time. A commercially available alternative capable of accommodating multiple lung slices operates by applying a negative pressure vacuum to deform an elastic membrane around a rigid post. However, we found that indenter posts with integrated ball bearings improved hysteresis, stretch homogeneity, and inter-cycle repeatability compared to other designs using grease to reduce friction, which can also cause heat-induced cell damage (Arold et al., 2009). Moreover, the flexframe design introduced here is considerably more economic, easy to build, reusable, and customizable with significantly lower intra-well variance. Although stretcher selection is generally dictated by application and familiarity, our device as described above is ideal for higher throughput testing of acute exposures, either pathologic or therapeutic, under tightly-regulated, physiologic stretching conditions.

This platform is uniquely appropriate for investigating mechano-inflammatory interactions, such as those underlying COPD. Biomechanical forces are known to facilitate emphysema progression (Mishima et al., 1999; Kononov et al., 2001; Yi et al., 2016) along with inflammatory stimuli (*i.e.*, cigarette smoking) that weaken and predispose tissue to failure (Suki et al., 2003). Yet, there is a paucity of data describing their relationship. CSE has been used with *in vitro* (Nana-Sinkam et al., 2007; Stringer et al., 2007; Thaikoottathil et al., 2009; Farid et al., 2013; Ballweg et al., 2014; Chen et al., 2014) and small animal (Chen et al., 2009, 2010; Hanaoka et al., 2011; Lee et al., 2012; He et al., 2015; Chai et al., 2016) models of cigarette smoke exposure given its relatively short incubation time and similarity to pathophysiology *in vivo*. As a proof of concept, we used our system to characterize the PCLS response to acute CSE exposure under various stretch patterns, simulating cigarette smoke inhalation during tidal breathing-like conditions.

IL-1β and MMP-1 expression are often upregulated in patients with COPD (Imai et al., 2001; Ostridge et al., 2016), while MT1-MMP and TIMP1 imbalance can lead to improper lung tissue maintenance (Vandenbroucke et al., 2011; Woode et al., 2015). We observed that the interaction between stretch pattern and CSE exposure had a significant effect on these markers. IL-1β increased with CSE and showed the most robust response to stretch, whereas the enzymes MT1-MMP and MMP-1 and the inhibitor TIMP1 could be either up- or down-regulated by CSE depending on the level of stretch. Interestingly, the low stretch group showed the greatest tissue content of MT1-MMP and MMP-1 when CSE was present, and conversely when it was absent for TIMP1, suggesting this stretch pattern may be most sensitive to an acute exposure. Additional silver staining revealed similar regulatory effects on protein species across a range of molecular weights (Supplementary Figure S2 and Supplementary Table S1). While not a comprehensive model of COPD, the stretch-dependent response to acute CSE exposure observed here suggests a role for mechanotransduction in modulating regional inflammation and enzyme burden on the alveoli. One may speculate this could further exacerbate structural disease heterogeneity and emphysema progression, particularly in tissue experiencing abnormal stretch (Mishima et al., 1999; Suki et al., 2003; Bhatt et al., 2016, 2017; Bodduluri et al., 2017; Mondoñedo and Suki, 2017). In any case, these findings show a clear and definitive difference in PCLS response to an acute exposure between cyclically stretched and unstretched conditions, highlighting the need to provide a comparable dynamic environment as experienced by the lung during tidal breathing *in vivo*.

There are some limitations of this study. (1) Bathing the lung slices directly in media simultaneously exposes all cell types to CSE, whereas exposure to inhaled cigarette smoke initially occurs at the airway and alveolar wall interfaces primarily involving epithelial cells. This is an inherent limitation of the PCLS design. Similarly, the MTS analysis does not specify local tissue viability, but could be extended with immunohistochemistry to verify cell origin. (2) The low-melt agarose is likely incompletely removed despite frequent media changes after slicing as in previous studies (Tepper et al., 2005; Sanderson, 2011; Davidovich et al., 2013b), which could affect the apparent stiffness and residual area of the lung slice. Thus, excised lungs were carefully prepared in the same manner each time to minimize disparities between animals. (3) The lack of circulation in the PCLS limits the study of chemotactic factors, including neutrophil recruitment, which participate in the inflammatory response to cigarette smoking (van der Vaart et al., 2004). (4) While this platform does not facilitate real-time imaging, flexframes are easily removed to visualize lung slices immediately after stretching. (5) Although the deformation provided by the equibiaxial stretching is not 3-dimensional uniform expansion, cells experience physiologically appropriate stretch as the aspect ratio is approximately 1 to 16.

In summary, we demonstrated the feasibility of using this device to perform high-throughput testing of an acute exposure under tightly-regulated, cyclic stretching conditions. We showed that pro-inflammatory and enzyme expression-related effects of acute exposure to cigarette smoke extract are stretch-dependent. These findings identify a fundamental difference between static and tidal breathing-like conditions in precision-cut lung slices. Additional studies in PCLS are required to determine whether mechanotransduction could be a key mediator in COPD disease pathogenesis and progression.

## Data Availability Statement

All datasets generated for this study are included in the article/Supplementary Material.

## Ethics Statement

This animal study was reviewed and approved by the IACUC of Boston University.

## Author Contributions

JM designed the stretcher and experiments, carried out studies, analyzed the data, and wrote the manuscript. EB-S carried out the biochemical assays and analyzed the data. SB analyzed the data. AS analyzed the data and designed the stretcher. KN, KC, AS, WO, and SR-M carried out the experiments. RK designed the experiments. JI designed the stretcher. BS designed the stretcher and experiments, analyzed the data, and wrote the manuscript.

## Conflict of Interest

BS and RK are co-owners of Mechanobiologix, LLC. Mechanobiologix received NIH funding to develop multi-purpose stretcher devices. The remaining authors declare that the research was conducted in the absence of any commercial or financial relationships that could be construed as a potential conflict of interest.
