# Supplementary material for: A High-Throughput System for Cyclic Stretching of Precision-Cut Lung Slices During Acute Cigarette Smoke Extract Exposure
Source: Front Physiol. 2020 Jun 5;11:566. doi: 10.3389/fphys.2020.00566 (PMC7326018; doi:10.3389/fphys.2020.00566)
Supplement: Supplementary file 1 [file Data_Sheet_1.PDF]

Supplemental Figure S1

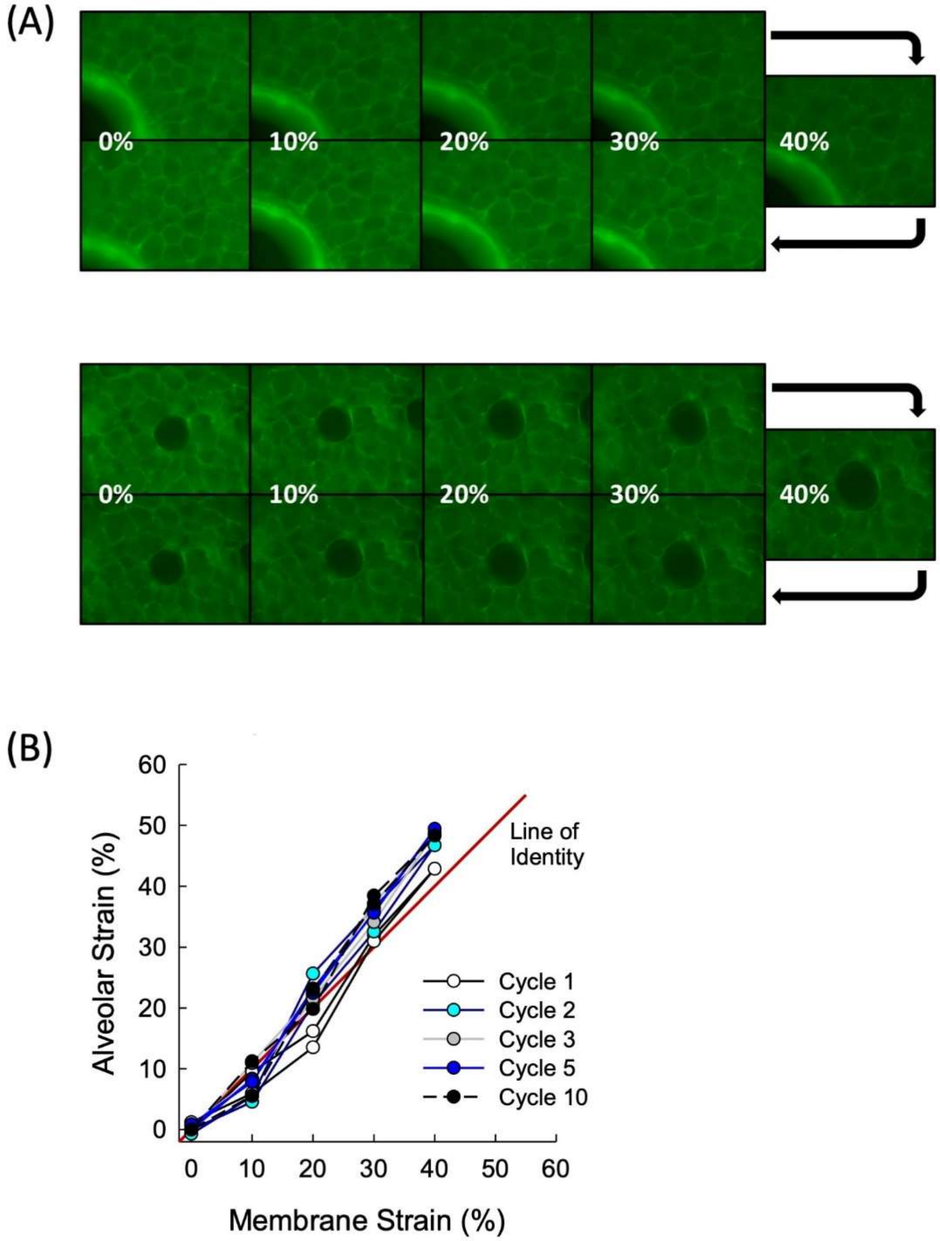

Supplemental Figure S1. Initial pilot studies confirmed that local tissue stretch matched that of the elastic membrane using the attachment technique detailed in the Methods section. Since the multi-well stretcher does not facilitate real-time imaging during stretch, a larger single-well version of this device (as described in Imsirovic et al. PLoS One 2015) was used to track changes in the lung parenchyma during pseudo-static stretch-relaxation cycles. Lung slices were prepared under identical conditions. Representative fluorescent images of the parenchyma near large (top panel) and small (bottom panel) airways are

shown (Fig. S1A). We found that changes in area strain for individual alveoli closely followed the prescribed membrane strain during consecutive stretch cycles (Fig S1B).

Supplemental Figure S2

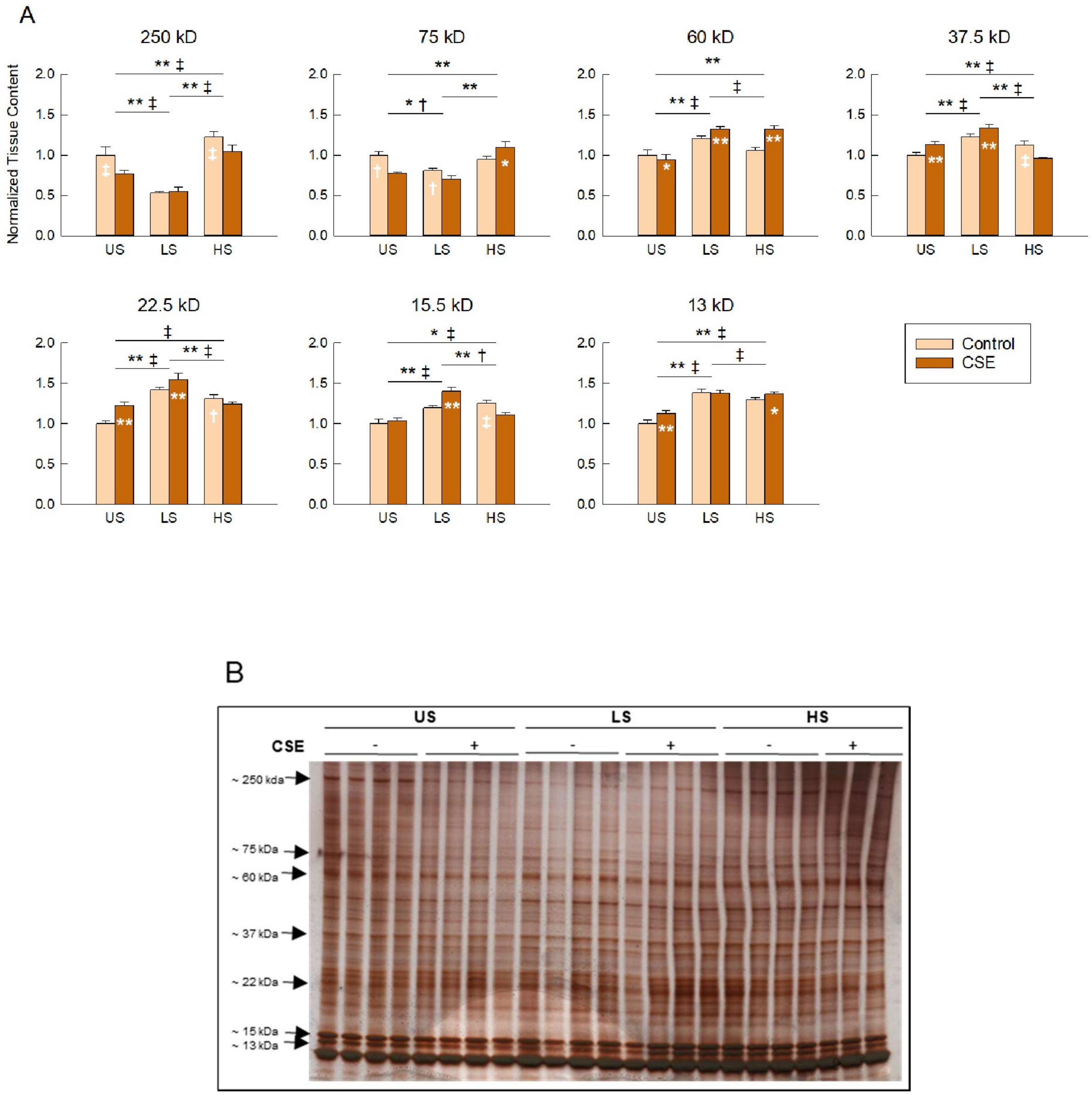

Supplemental Figure S2. (A) As described in the main text, a BCA colorimetric protein assay kit (Pierce, Thermo Scientific) was used to determine the protein concentrations of the homogenized tissues. Equal amounts of protein (~1.8 μg) from each sample were separated via SDS-PAGE. Gels were stained using the Silver Stain Plus Kit (BioRad) for protein species visualization in total protein samples, and quantitative densitometry was

performed. Two-Way analysis of variance (ANOVA) revealed stretch pattern, CSE, and their interaction had significant effects on protein species across a range of molecular weights. Data (N=6-8) are shown as normalized mean and SD (*,† indicate $p<0.05$ and **,‡ indicate $p<0.001$ for CSE and Control groups, respectively). (B) Representative silver staining of lung PCLS treated with (+) or without (-) CSE. US: unstretch; LS: low stretch; HS: high stretch.

Supplemental Table S1.

Weight (kD)	Effect of CSE	Effect of Stretch	Effect of Interaction
250	<0.001	<0.001	<0.001
75	0.010	<0.001	<0.001
60	<0.001	<0.001	<0.001
37.5	0.029	<0.001	<0.001
22.5	<0.001	<0.001	<0.001
15.5	0.015	<0.001	<0.001
13	<0.001	<0.001	<0.001

Supplemental Table S1. P-values for silver staining
